# Biomaterials for Tissue Engineering Applications and Current Updates in the Field: A Comprehensive Review

**DOI:** 10.1208/s12249-022-02419-1

**Published:** 2022-09-26

**Authors:** Alaa Emad Eldeeb, Salwa Salah, Nermeen A. Elkasabgy

**Affiliations:** Department of Pharmaceutics and Industrial Pharmacy, Faculty of Pharmacy, Cairo University, Kasr El-Aini Street, Cairo, 11562 Egypt

**Keywords:** Bioactive mineral fillers, Biomaterials, COVID-19, Smart polymers, Tissue engineering, 3D printing

## Abstract

Tissue engineering has emerged as an interesting field nowadays; it focuses on accelerating the auto-healing mechanism of tissues rather than organ transplantation. It involves implanting an *In Vitro* cultured initiative tissue or a scaffold loaded with tissue regenerating ingredients at the damaged area. Both techniques are based on the use of biodegradable**,** biocompatible polymers as scaffolding materials which are either derived from natural (e.g. alginates, celluloses, and zein) or synthetic sources (e.g. PLGA, PCL, and PLA). This review discusses in detail the recent applications of different biomaterials in tissue engineering highlighting the targeted tissues besides the *in vitro* and *in vivo* key findings. As well, smart biomaterials (e.g. chitosan) are fascinating candidates in the field as they are capable of elucidating a chemical or physical transformation as response to external stimuli (e.g. temperature, pH, magnetic or electric fields). Recent trends in tissue engineering are summarized in this review highlighting the use of stem cells, 3D printing techniques, and the most recent 4D printing approach which relies on the use of smart biomaterials to produce a dynamic scaffold resembling the natural tissue. Furthermore, the application of advanced tissue engineering techniques provides hope for the researchers to recognize COVID-19/host interaction, also, it presents a promising solution to rejuvenate the destroyed lung tissues.

**Graphical abstract:**

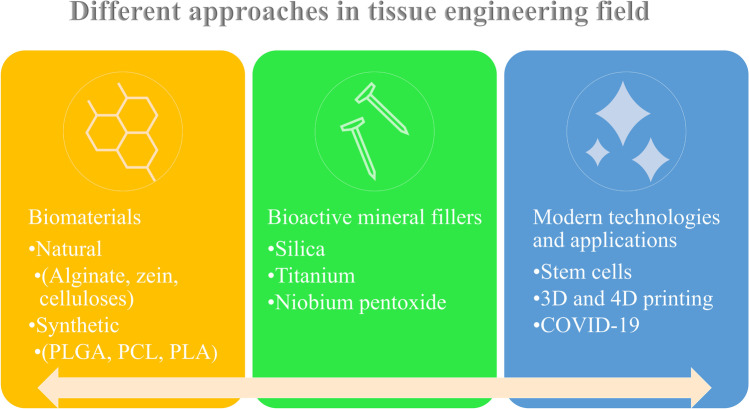

## Introduction

Tissue engineering is a field concerned with the development of functional tissues by the combination of cells, scaffolds, biomaterials, and biologically active ingredients. The main aim of tissue engineering is to fabricate functional constructs that restore and improve damaged tissue functions [1–9]. It was first introduced in the late 1980s in a meeting held by the National Science Foundation in the USA [10], while the first published paper that used the term *tissue engineering* as it is known today was in 1991 by a paper entitled “Functional Organ Replacement: The New Technology of Tissue Engineering” [11], while in 2008, the first completely tissue-engineered organ was a trachea transplanted in a 30-year-old woman to replace an end-staged damaged airway [12, 13].

Tissue engineering is divided into *ex vivo* tissue engineering and in *situ* tissue engineering [14, 15]. (i) *Ex vivo* tissue engineering involves the isolation of stem cells from the donor to be seeded on an external scaffold in a suitable environment in bioreactors to stimulate cell proliferation and differentiation into the desired tissue [16–19]. The produced tissue is then implanted into the desired tissue, so it must be of the same size and shape as the defect area, the scaffold degrades over time to permit the substitution with the newly regenerated tissues [20–23]. This approach presents scaffolds of good mechanical properties and allows the use of many biomaterials [24–28]; however, it requires sophisticated optimization of the conditions in the bioreactors to allow initial cell proliferation, high cost, donor site morbidity**,** and rejection of the implanted tissue may occur [29]. While (ii) *in situ* tissue engineering represents a simple and convenient solution that involves the pre-fabrication of a scaffold made from biocompatible biomaterials with a specific size and shape and its implantation directly into the required tissue without the need for prior seeding with cells, it relies on attracting the surrounding cells by promoting the host’s tissue regeneration [8, 14, 15, 30, 31]. As a result, it provides an immune-compatible alternative for the *ex vivo* approach so the rejection of the implanted scaffold is avoided [32]. Nevertheless, the regenerated tissue may suffer from poor mechanical properties as this approach lacks good control over cellular differentiation [33]. To be noticed, *ex vivo* tissue engineering is the only way to develop non-regenerating tissues (e.g. cardiac and neural tissues), yet, the optimization and reproducibility of cell seeding conditions are not easy [34, 35].

In this review, we will discuss the recent applications of different biomaterials (natural and synthetic) as well as the use of different bioactive mineral fillers for tissue engineering purposes. Furthermore, the review sheds the light on the current strategies and applications in the tissue engineering field like the use of stem cells which combat the limitations of conventional tissue engineering strategies. 3D and 4D printing techniques are discussed elaborating on the benefits gained from these current trends. The progress of the tissue engineering field was reflected in controlling some of the complications associated with COVID-19.

## Biomaterials in Tissue Engineering

**Fig. 1:**
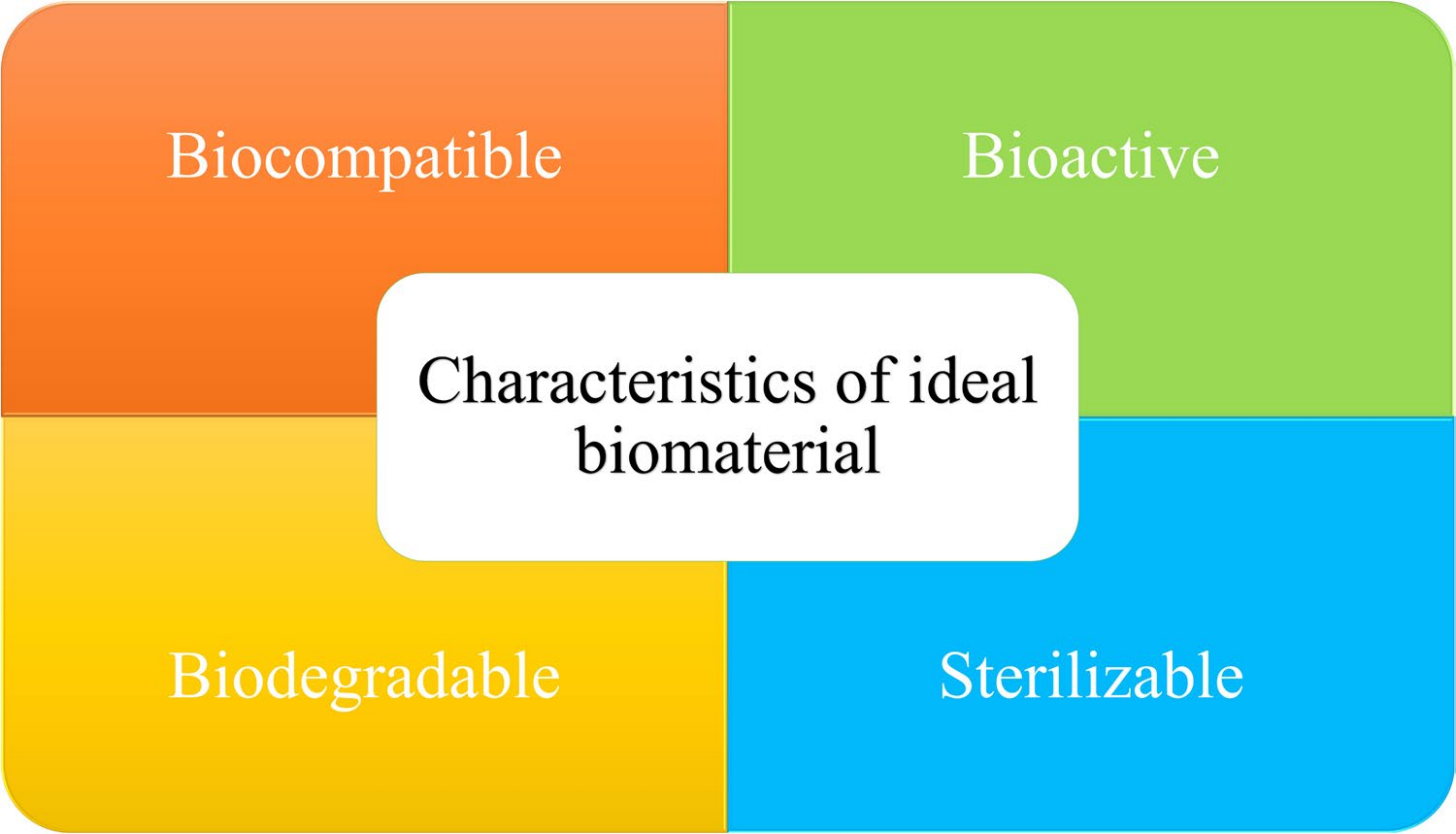
Characteristics of the ideal biomaterials in tissue engineering applications

The objective of tissue engineering is to repair and regenerate damaged tissues by different means. Biomaterials are defined as any material, construct, or surface that interacts with biological systems. It could be derived from natural sources or made synthetically, which are used for partial or full tissue replacement. Regardless of the origin, they must be biocompatible to avoid the induction of immune response besides being sterilizable to be safely incorporated into the host tissues, biodegradable to disappear from the tissue after fulfilling their function, and bioactive to stimulate tissue responses (Fig. 1) [36, 37]. Natural polymers (chitosan, gelatin, collagen, cellulose, alginates, etc.) are more preferred than synthetic ones (polylactide-co-glycolide (PLGA), polycaprolactone (PCL), polylactic acid (PLA), fibronectin, polyurethane, etc.) as they have higher biocompatibility, excellent biodegradability, and minimal toxicity [38]. Moreover, plant-derived biomaterials have been explored widely to replace animal-derived ones due to major concerns regarding variability, ethical and environmental issues, also, animal-derived products are of higher cost and need more extraction and purification processes [39].

### Natural Biomaterials

#### Alginates

Alginate is a natural polysaccharide derived from brown algae. It is composed of repeated units of β-1,4 linked D-mannuronic acid and L-guluronic acid in different ratios. The high percentage of D-mannuronic acid residues decreases the tackiness of alginate and stimulates immunogenic responses when presented to the body [40, 41]. Nevertheless, the high content of L-guluronic acid residues promotes the possibility of hydrogel creation via ionic crosslinking with divalent cations (e.g. Ca^2+^). The crosslinking occurs through the interaction of each Ca^2+^ ion with two opposing L-guluronic acid blocks, forming a crosslinked network identified as “egg-box” conformation [42–45]. Alginate is listed as a generally regarded safe (GRAS) substance by the FDA [46] with outstanding properties. It is considered a perfect biomaterial for tissue engineering owing to its biodegradability, biocompatibility, hydrophilicity, affordability, and elasticity [47–49].

Alginates provide a moist environment and decrease bacterial infections, so they possess high patient acceptability in wound healing. They promote the release of specific cytokines which aid in accelerating the wound healing process [50, 51]. Additionally, they boost re-epithelialization and granulation tissue formation which consequently promote wound healing [52]. Literature includes several studies highlighting the beneficial effect of using alginates in formulating different dosage forms applied for wound healing. Among those dosage forms are hydrogels [53], sponges [54], membranes [55], nanofibers [56], foams [57], and films [58].

In the same context, Ma *et al.* fabricated a wound dressing by combining the wound healing effect of carboxymethyl chitosan and sodium alginate, in addition to the antibacterial effect of Ce3 + ions. Hybrid spheres of carboxymethyl chitosan and sodium alginate were developed by their crosslinking with Ce^3+^ using the electrostatic spray technique. Crosslinking with Ce^3+^ improved the stability of the dressing compared to non-crosslinked sodium alginate droplets, where it showed gradual swelling till 12 h while sodium alginate droplets completely degraded within 1 h. Cell viability study on mouse fibroblasts (cell line L929) showed good biocompatibility. The cross-linked spheres showed a pronounced inhibitory effect on *Staphylococcus aureus* and *Escherichia coli* due to the release of Ce^3+^, which is highly favored in the wound healing process as bacterial growth is the main reason for delayed wound healing. *In vivo* study on Kunming mice showed that the crosslinked spheres facilitated complete healing of the induced wound within 10 days [59].

A recent study succeeded to prepare three-dimensional (3D) printed aerogel scaffolds composed of a mixture of alginate and hydroxyapatite for bone tissue engineering. Cell viability percentage was evaluated using mouse embryo fibroblast (BALB/c3 T3) cells after 24 and 48 h incubation of cells with the aerogel against positive control of the cells with a medium. Cell viability results showed nearly 100% viability indicating non-cytotoxicity of the prepared scaffolds. The formed scaffolds exhibited high porosity which enhanced the cellular adhesion along with cellular proliferation after 13 days of incubation [60].

Moreover, Eldeeb *et al.* fabricated 3D nanocomposite alginate hydrogel containing pitavastatin nanovesicles as a bioactive wound dressing. The nanocomposite alginate hydrogel was prepared simply by the cross-linking of the nanovesicular alginate gel by 0.2 M CaCl_2_. The hydrogel showed a sustained drug release for 7 days with an initial drug release of 47.30 ± 0.23% after 24 h and a maximum water absorption capacity of 910.60 ± 106.03% after 21 days. *In vivo* evaluation of the nanocomposite alginate hydrogel, plain alginate hydrogel, and drug suspension on surgically-induced skin wounds in Mongrel dogs proved the superiority of the fabricated nanocomposite hydrogel in the wound healing process as the wound gap almost disappeared with the formation of a complete skin structure (confirmed by a histological evaluation) within 4 weeks. In contrast, groups treated with plain alginate hydrogel and drug suspension, respectively, showed incomplete development of skin structures. While the control group (untreated) showed incomplete wound healing, in addition, a clear wound gap was seen [43]***.***

#### Celluloses

It is the most abundant and influential organic biopolymer on earth [61]. It comprises the main structural component of the plant cell wall, also, it could be extracted from some bacteria and algae [62]. It is a linear (non-branched) polysaccharide composed of repeated units of β-D-glucose linked together through 1,4 links [63]. It is characterized by being eco-friendly and biodegradable with high tensile strength. Cellulose is usually obtained from wood pulps and cotton for industrial purposes. It contributes to different applications like paper and textile production [64] as well as pharmaceutical [65], cosmetics [66], and tissue-engineering applications [67].

Mahendiran *et al.* prepared 3D porous cellulose scaffolds derived from *Borassus flabellifer* fruit. The obtained porous 3D scaffolds were called *Borassus flabellifer* endosperm-derived cellulosic scaffolds. Two scaffolds were fabricated and compared: one is a cellulosic scaffold and the other is the hybrid cellulose-chitosan scaffold. To prepare the hybrid scaffolds, chitosan solution in acetic acid (1%, w/v) was blended with the same volume of *Borassus flabellifer* decellularized sample then filled into 24 well-plates and freeze-dried for 48 h to yield a 3D porous hybrid scaffold. The cellulose scaffold exhibited a swelling capacity of 2067.59 ± 36.88%, while the hybrid scaffolds showed a swelling capacity of 997.83 ± 68.69% after 24 h of incubation with PBS. Both scaffolds revealed good porosity with pore size ranging from 50 to 200 µm besides controlled degradation with 59.21 ± 0.6% and 47.80 ± 0.45% weight loss after 21 days of incubation in Dulbecco’s Modified Eagle’s medium–high glucose media for cellulose scaffold and hybrid scaffold, respectively. Cell viability and L929 cell attachment revealed a significant increase in cell count after 5 days with improved cell attachment for the hybrid scaffold compared to the cellulose-based scaffold. In conclusion, both scaffolds are suitable candidates for tissue engineering applications. Nevertheless, this study lacked *in vivo* studies, and long-term stability [39].

Hospodiuk-Karwowski *et al.* produced bacterial cellulose from *Komagataeibacter hansenii* which was then carboxymethylated by two-step-reaction. The produced carboxymethyl cellulose carrying a negative charge was then cationized by the syringing of the diluted carboxymethyl cellulose to a pool of 1% chitosan solution while homogenizing; afterwards, the resultant dispersion was centrifuged, and the positively-charged sedimentary pellets were collected. Mixing of the positively and negatively charged celluloses was assessed to prepare different bioinks for the fabrication of 3D printing of scaffolds. Rheological assessment of the prepared bioinks was evaluated using a strain-controlled rheometer and revealed a shear-thinning behavior that is suitable for 3D printing purposes. Cell viability was tested by the incubation of the prepared bioinks with rat heart microvessel endothelial cells for a week and the results demonstrated > 80% viability. This research presents a tailored combination that can be customized to meet specific needs for tissue engineering [68].

With the expansion of nanoscience, many researchers are focusing on the production and modification of nanocellulose that can be applied to versatile treatments [69]. Nanocellulose is available in many forms, i.e. nanocrystalline and nanofibrillated cellulose which is obtained by degrading plant fibers, bacterial nanocellulose which is produced by specific bacteria and characterized by high water affinity comparable to that of hydrogels [70], and bacterial cellulose whiskers which is the hydrolysate of bacterial cellulose [71].

Volz *et al.* utilized bacterial nanocellulose derived from *Gluconacetobacter xylinus* to fabricate artificial adipose tissue constructs to facilitate deep wound healing. The prepared system showed accelerated adipogenic differentiation and facilitated the vascularity of human vascular endothelial cells, thus, accelerating wound healing. This research presented bacterial nanocellulose as a promising candidate for vascularized adipose tissue engineering [72].

One study highlighted the beneficial effect of nanofibrillated cellulose in bone tissue engineering. The authors managed to prepare 3D scaffolds made up of nanofibrillated cellulose/cyclodextrin blend loaded with raloxifene hydrochloride by freeze-drying. Two types of cyclodextrins were tried; beta-cyclodextrin and methyl-beta-cyclodextrin. Cyclodextrins were used to enhance drug solubility [73]. Scaffolds displayed high porosity > 90% with good mechanical properties. The prepared scaffold possessed controlled drug release for 480 h with low initial burst release when using beta-cyclodextrin compared to methyl-beta-cyclodextrin which might be ascribed to the lower solubility of the former. Also, *in vitro* cell viability testing demonstrated improved cell adhesion and proliferation with significant induction of alkaline phosphatase (ALP) production and calcium ions accumulation [74].

#### Zein

Zein is a plant protein with molecular weight varying from 22 to 27 kDa [75], it is considered the main storage protein of corn. It belongs to the class of prolamins which is mainly composed of hydrophobic amino acids responsible for its characteristic hydrophobicity [76]. Its biodegradability, biocompatibility, availability, low cost as well as safety (classified as GRAS by the FDA) made it a suitable candidate for drug delivery applications [77]. Among those applications, zein was utilized for the fabrication of scaffolds for tissue engineering [78–82], vaccine delivery [83], DNA transfection [84], oral delivery of peptides and proteins [85], and colon-specific drug delivery [86]. The rising potential of the specific use of zein in bone tissue engineering refers to its perfect membrane forming ability (osteoconductivity), optimum biodegradability, suitable mechanical properties (toughness, flexibility, and compressibility), resistance to degradation by microbial enzymes, and intrinsic anti-oxidant activity [87, 88]. Though it lacks a cell proliferative effect, so blending zein with bioactive substances (e.g. bioceramics, calcium phosphates, and osteoinductive drugs) is preferred to support the proliferation and differentiation of bone cells [82, 89, 90].

Eldeeb *et al.* formulated dual zein *in situ* forming implants for bone regeneration loaded with pitavastatin calcium and tedizolid to impart osteogenic and antibacterial properties to the implant, respectively. A titanium-doped bioactive glass was added to enhance the bone proliferative effect of the formulated implants, also, sodium hyaluronate was included as a porogenic agent to induce the porosity required for cellular infiltration and proliferation. The fabricated implant showed a sustained release of both drugs for 28 days. *In vivo* studies on Sprague Dawley rats demonstrated a significant bone regenerating effect [82]. This study succeeded to introduce zein as a promising implant matrix; however, more studies and investigations should be conducted to optimize the fabricated implant to be applicable for large-sized bone defects.

Mariotti *et al.* used the electrospinning technique to produce fiber mats of zein blended with either non-doped or copper-doped bioactive glass. A degradation study for 14 days in Dulbecco’s Modified Eagle’s medium showed a gradual formation of halite crystals with gradual loss of the fibrous structure of scaffolds; however, some fibers contained bioactive glass at the end of the study which suggested a sufficient strength of the scaffolds*. In vitro* cell viability studies for zein fiber mats loaded with copper-doped bioactive glass on human osteosarcoma cell line MG-63 as well as on mouse muscle cell line C2C12 revealed improved cell proliferation up to 61 *versus* 59% after 7 days, respectively. The antibacterial activity was assessed by incubating the scaffolds with *Staphylococcus aureus* and *Escherichia coli* for 3 days and the results showed inhibited bacterial growth when using the zein fiber mats loaded with copper-doped bioactive glass compared to plain mats as well as zein fiber mats loaded with non-doped bioactive glass, which might be ascribed to the antibacterial activity of copper ions [91].

Nanofibrous scaffolds comprising zein/PCL/collagen and containing *Aloe vera* and ZnO nanoparticles were prepared via electrospinning technique. The developed nanofibers showed a controlled ZnO release of up to 70% after 28 days, suitable thermal stability, and good mechanical properties. Additionally, the developed fibers showed excellent cytocompatibility and enhanced cellular adhesion when incubated with human gingival fibroblasts when compared to plain nanofibers lacking the addition of *Aloe vera* and ZnO nanoparticles. Also, they showed good antibacterial activity with inhibition zones up to 19.23 ± 1.35 and 15.38 ± 1.12 mm against *Staphylococcus aureus* and *Escherichia coli*, respectively*.* This research presented the fabricated nanofibrous scaffold as a bioactive wound dressing [78].

Tavares *et al.* fabricated composite films of chitosan/zein incorporating ellagic acid for treating skin infections and enhancing skin recovery. The formed films showed a suitable thickness ranging from 133 ± 51 to 283 ± 75 µm and a high percentage of water uptake between 114.44 ± 8.07 and 227.94 ± 25.88% after 48 h as well as a sustained drug release up to 6% after 48 h. Furthermore, films demonstrated *in vitro* antibacterial effects against *Staphylococcus aureus* and *Pseudomonas aeruginosa* [92]. Further *in vivo* investigations on experimental animals should be carried out to investigate the safety and efficacy of the proposed formulation.

Nanofiber membranes of cellulose acetate and zein mixtures were developed to incorporate sesamol for diabetic wound healing. The measured water contact angles before and after the addition of sesamol to the membranes were 74.5° and 36.5°, respectively which proved quick water infiltration due to the hydrophilicity imparted by sesamol. *In-vitro* release of sesamol from the nanofiber membrane in ethanol revealed a burst release with around 70% released within the first 20 min and extended up to 90% at 120 min, also, sesamol release in water showed similar results. *In-vivo* study on diabetic male mice showed enhanced wound healing with the induced production of collagen-III and stimulation of transforming growth factor-β expression (an important marker in wound healing), in addition to that, it reduced the expression of many inflammatory mediators providing a new promising wound dressing [93].

Arango-Ospina *et al.* fabricated a multifunctional bone regenerating scaffold, where authors targeted to combine the biocompatibility and biodegradability of zein along with the antibacterial activity of Manuka honey. Scaffolds were prepared by coating bioactive glass with both zein and Manuka honey. Scaffolds showed good mechanical properties with compressive strength of 0.14 ± 0.05 MPa revealing the beneficial effect of the added coat in improving the brittleness of uncoated bioactive glass scaffolds. The porosity of the scaffolds was calculated to be 95.6 ± 0.3% for uncoated scaffolds and 77. ± 3.0% for the coated scaffolds with zein and 20 wt.% Manuka honey. The evaluation of bioactivity was performed in simulated body fluid (SBF) by observing the development of hydroxycarbonate apatite layer on the surface of scaffolds via scanning electron microscope (SEM) and Fourier Transform Infrared (FTIR) analysis. Results declared that the coated scaffolds showed a growing apatite-like layer on their surfaces after 1 week of incubation while the uncoated ones developed the apatite layer after 1 day only. The release behavior of Manuka honey from the scaffold showed a very fast release within the first hour due to the hydrophilicity of the honey besides the low encapsulation percentage of the honey inside the zein matrix. Assessment of the antibacterial activity of the scaffolds containing 20 wt.% Manuka honey on *Staphylococcus aureus* revealed the greatest antibacterial activity when compared to other samples with lower wt.% of Manuka honey. Further studies and additional attention should be given to improve the encapsulation of Manuka honey inside zein coating and further assessments of the *in-vivo* performance of the developed scaffolds should be investigated [94].

A fibrous scaffold of zein/chitosan/polyurethane composite linked with functionalized multiwalled carbon nanotubes was developed as a bone regenerating scaffold. The formed nanofibers showed a uniform small diameter averaging ≈ 126 nm and viscosity equal to 88.93 ± 0.61 cp. The surface of the fibrous scaffold was completely covered with apatite-like layer with large uniform CaCO_3_ crystals after immersion in SBF for 5 days. Antibacterial activity was assessed by measuring the inhibition zone after incubating the scaffold for 12 h with various bacterial strains (*Escherichia coli*, *Staphylococcus aureus*, *Micrococcus luteus*, and *Staphylococcus epidermidis*). Results revealed high death rates of these bacteria where clear inhibition zones were detected. Cell viability testing on MC3T3-E1 cells showed high cellular adhesion and proliferation over 7 days with enhanced ALP production after 10 days of incubation. [95]. However, the study lacked *in vivo* evaluation of the prepared scaffolds.

More studies highlighting the use of the afore-mentioned natural biomaterials are summarized in Table I.

**Table I:** An Overview of the Applications of Natural Biomaterials in Tissue Engineering

Drug	Composition	Fabricated dosage form	Targeted tissue & application	Key findings	References
Cannabidiol	Alginate crosslinked with Zn^2+^ ions	Hydrogel dressing	Skin wounds	• Drug release studies in 0.1% (w/v) Tween® 80 solutions in phosphate buffer saline (PBS; pH = 7.4) demonstrated that after the immersion of the hydrogels for 24 h, the cannabidiol release from different formulations ranged from 49–68% then reached a plateau	[96]
				• Excellent swelling ratio up to 50% after 5 h as well as acceptable rheological properties	
				• Anti-oxidant behavior of the hydrogel was confirmed by the study	
				• The antibacterial activity against *Staphylococcus aureus* (ATCC 25,923) and *Escherichia coli* (ATCC 25,922) was proved	
				• *In vitro* cell viability studies on human umbilical vein endothelial cells and mouse embryonic fibroblast cells (NIH 3T3) showed promoted cell proliferation with excellent biocompatibility	
				• *In-vivo* studies on Sprague Dawley rats revealed enhanced wound healing indicated by stimulated collagen production, granulation tissue**,** and blood **vessel** formation compared to the control group treated with saline solution	
Bovine serum albumin	Alginate/gelatin-methacryloyl blend coated with nanoapatite (thickness of 10–80 μm)	3D printed composite scaffolds with hollow channel structure	Bone defects	• A porosity of 78.7 ± 3.2% and a sustained release of protein for 28 days	[97]
				• *In vitro* studies on osteoblasts (isolated from Sprague–Dawley rats) indicated good biocompatibility, improved cellular interaction, and enhanced proliferation after 7 days of incubation	
				• *In vivo* study on adult male Sprague Dawley rats showed almost complete healing of the defect where the gap was completely filled with new bone tissues after 12 weeks of implantation	
Indomethacin	Zein/ethyl cellulose	Electrospun nanofibrous composites	Skin wounds	• The formed composite showed enhanced water stability up to 56 h	[98]
				• Excellent mechanical properties	
				• The composite succeeded to sustain the drug release (≈50%) for 56 h presenting it as a promising wound dressing	
Aloe vera/ZnO nanoparticles	Zein/PCL/collagen	Nanofibrous scaffolds	Skin wounds	• The developed nanofibers showed a controlled ZnO release of up to 70% after 28 days, suitable thermal stability, and good mechanical properties	[99]
				• Excellent cytocompatibility and enhanced cellular adhesion when incubated with human gingival fibroblasts when compared to plain nanofibers lacking the addition of Aloe-vera and ZnO nanoparticles	
				• Good antibacterial activity with inhibition zones up to 19.23 ± 1.35 and 15.38 ± 1.12 mm against *Staphylococcus aureus* and *Escherichia coli*, respectively	

### Synthetic

#### PLGA

Poly (lactic-co-glycolic acid) is a linear aliphatic copolymer composed of monomers of lactic acid and glycolic acid [100–102]. It could be synthesized by the polycondensation of lactic and glycolic acids, which produces PLGA with low molecular weight and relatively high molecular weight distribution. Another method is the ring-opening polymerization of lactide and glycolide, which gives a higher molecular weight PLGA with a consistent molecular weight distribution [103]. PLGA is a biocompatible, biodegradable, FDA-approved copolymer [104] with an adjustable degradation rate (depending on the ratio of lactide to glycolide). It degrades by hydrolytic de-esterification to its monomers and then is eliminated naturally by the body [105]. However, the usage of PLGA alone is not common in tissue regeneration as it lacks osteoinductive activity and demonstrates inadequate mechanical properties for use in load-bearing sites, so it is usually used in a combination with other materials [105].

The literature presented one study demonstrating the combination of hybridized PLGA with hydroxyapatite nanoparticles to solve the lack of osteogenicity of plain PLGA scaffolds. The authors produced 3D-printed porous scaffolds for bone regeneration via fused deposition modeling. The fabricated scaffolds displayed excellent cytocompatibility with 100% viability when seeded with human adipose-derived stem cells and human bone marrow stromal cells after 7, 14, and 21 days of incubation. ALP was detected after 14 days of incubation with significant Ca^2+^ deposition after 21 days indicating good osteogenic ability on both types of cell lines. *In-vivo* biocompatibility was assessed by observing the formation of fibrotic bands after subcutaneous implantation of the scaffolds in Wistar rats. Additionally, the site of application was assessed histopathologically at 1^st^- and 4^th^-week post-implantation using a scoring system, where the scaffolds recorded a slightly inflammatory response revealing good biocompatibility [106].

Layered double hydroxides are among the mineral particles used in tissue engineering to promote osteogenesis. Shokrolahi *et al.* intercalated atorvastatin in Mg/Mn-layered double hydroxides to fabricate layered double hydroxide/PLGA composite by electrospinning for hard tissue engineering. The system showed 60% drug loading with an initial burst release of nearly 40% after 1 day and a plateau for 14 days. Assessment of the *in-vitro* bioactivity of the scaffolds incubated with adipose-derived mesenchymal stem cells showed a high ALP production (an early marker of osteogenic differentiation) of 280 lu/mg total protein content compared to the unloaded PLGA group signifying the osteogenic ability of the prepared scaffolds. Alizarin Red staining of the cells (a late-stage marker of osteogenic differentiation) showed intense staining after 14 days indicating that the cells entered the mineralization phase 14 days after cell seeding [107].

Zare *et al*. developed nanocomposites based on the combination of PLGA with metal-based nanostructures and discussed their biomedical applications. Metal-based nanostructures were indicated to impart antimicrobial effects and osteoinductive properties to PLGA [108]. Some examples of their use in tissue engineering applications include the following: (i) Selenium/PLGA combination which showed good antibacterial activity against Gram-positive bacteria commonly causing orthopedic infections [109]. (ii) Fe_3_O_4_/PLGA fibrous scaffolds that are used to prepare bone regenerating scaffolds [110]. (iii) TiO_2_-nanotubes/PLGA combination increased cell viability significantly more than pure PLGA [111]. (iv) Ag nanoparticles/PLGA introduced antibacterial and bone regenerative properties to the scaffold [112].

Putri *et al.* blended PLGA mesh with collagen sponge layer in the presence of ice particulates to prepare scaffolds for cartilage tissue engineering. The fabricated scaffold showed 99.1 ± 0.4% porosity with large (441 ± 52 nm) and small pores (50 ± 19 nm) as well as good mechanical strength (compressive strength > 8 kPa). Seeding bovine articular chondrocytes into the scaffold showed a promoted cell proliferation after 7 days which was assisted by the interconnected porous structure of the scaffold. Authors implanted the scaffold in the back of nude mice for 8 weeks, the results proved homogeneous distribution and growth of cells within the scaffold with uniform deposition of cartilaginous matrices [113].

#### PCL

Polycaprolactone (PCL) was firstly synthesized by Carothers and Hill in the 1930s [114]. It is a biodegradable, aliphatic, semi-crystalline polyester that could be synthesized by various methods [115, 116] with different molecular weights (14,000–100,000) and diverse properties [117]. It has a melting range of 59–64°C and a glass transition temperature of − 60°C, so it exhibits reasonable toughness and mechanical strength at body temperature. PCL possesses the slowest degradation rate ranging from months to years between the other polyesters due to the presence of repeating five hydrophobic moieties of -CH_2_ [118, 119]; however**,** the degradation rate could be tailored by varying its molecular weight, degree of crystallinity as well as the degradation conditions [120]. PCL is approved by the FDA to be used in different products [121] and has been extensively used in tissue engineering due to its biocompatibility, tunable biodegradability, good strength, ease of synthesis, facile modification into different shapes due to low melting point, less acidic byproducts when compared to other polyesters, and very high drug permeability [120]. However, its slow degradation rate [122], low cell adhesion [123], and its non-osteogenic properties provoked the preparation of different composites based on the combination of PCL with other biomaterials.

Kumar *et al.* developed biodegradable scaffolds with high mechanical strength (≈ 92 MPa) composed of PCL, polyglycolic acid (50% was the optimum), and beta**-**tricalcium phosphate (20%) by solvent casting method, followed by compression molding and sintering. The added polyglycolic acid enhanced the mechanical strength of the scaffold besides decreased its degradation time where nearly 40% of the scaffold degraded after 24 weeks. While beta tricalcium phosphate was added as a bioactive component to enhance bone regeneration. The developed scaffolds showed good porosity [124]. More investigations to assess the *in vitro* or *in vivo* osteogenic ability of the scaffold should be conducted.

Composite PCL electrospun fibrous scaffolds were excessively studied in the literature. In one study, Fadaiea *et al*. combined nanofibrillated chitosan with PCL at different concentrations to fabricate nanocomposite fibrous scaffolds by electrospinning. The prepared scaffolds showed good wettability, enhanced tensile strength, besides improved cell adhesion and proliferation, when tested on normal human dermal fibroblast cell lines [125]. In the same context, electrospun nanocomposite scaffolds were fabricated via the electrospinning of the PCL/gelatin blend followed by its treatment with nanohydroxyapatite (1%) for various periods. Scaffolds resulting after 20-min treatment showed a fiber diameter of 615 ± 269 nm and pore size of 4.7 ± 1.04 μm. Investigations using FTIR spectroscopy and thermogravimetric analysis assured the presence of nanohydroxyapatite over the surface of the scaffold. Moreover, *in vitro* cell viability studies showed enhanced human osteoblast proliferation and improved cellular attachment [126]. Another study succeeded to prepare electrospun nanofibrous PCL scaffolds loaded with silver-doped hydroxyapatite to promote wound healing and treat associated bacterial infections. Results confirmed the significant antibacterial activity exerted by the scaffolds which was proportional to the concentration of the silver. Besides the antibacterial efficiency, the prepared scaffolds managed to enhance human fibroblast proliferation significantly [127].

El-Habashy *et al.* prepared nanoparticulate hybrid hydroxyapatite/PCL scaffolds using the direct emulsification-solvent evaporation method. Evaluation of the bioactivity on mesenchymal cell proliferation proved that the hybrid scaffold showed enhanced osteogenicity and biocompatibility when compared to the plain hydroxyapatite nanoparticles [128].

Different authors utilized 3D printing techniques in fabricating PCL scaffolds for tissue regeneration. Alemán-Domínguez *et al*. blended microcrystalline cellulose (2, 5, and 10% w/w) with PCL to yield 3D printed scaffolds. Results concluded that the scaffold containing 2% microcrystalline cellulose improved the mechanical strength and porosity of the scaffold and significantly promoted the proliferation of sheep bone marrow cells compared to the other concentrations [129]. Park *et al.* blended tonsil-derived mesenchymal stem cells with PCL/beta-tricalcium phosphate to prepare a 3D printed prosthesis for mandible osteogenesis. A pilot animal study on New Zealand rabbits proved the success of the prepared scaffolds in promoting osteogenesis [130]. Navaei *et al.* combined 3D printed PCL mesh with gelatin scaffolds to generate a hybrid porous scaffold for diaphragm regeneration with enhanced mechanical strength, flexibility, and cellular adhesion by the addition of gelatin. Biocompatibility of the prepared scaffold was evaluated in Bagg Albino mice for 20 days and the study proved the superiority of the prepared PCL/gelatin scaffold compared to the plain PCL scaffold regarding cellular behaviour and attachment. The prepared hybrid scaffolds presented a successful strategy for diaphragm regeneration [131].

The use of PCL was not restricted to bone and skin tissue regeneration only; however**,** it was used to prepare constructs for cartilage [132], skeletal muscles [133], cardiovascular [134], and nerve regeneration [135].

#### PLA

PLA, known as polylactide, is a synthetic FDA**-**approved biodegradable, biocompatible polymer of lactic acid used for tissue engineering. Lactic acid is a naturally occurring organic acid produced by the fermentation of sugars derived from natural sources such as corn, wheat**,** and sugarcane. PLA is synthesized chemically by various methods, all of which take a long polymerization time and require the use of catalysts under controlled conditions (temperature, pressure**,** and pH) [136–138]. It degrades to its monomers (lactic acid) and then finally into CO_2_ and water. It has some drawbacks such as limited cell adhesion, slow degradation, and strong hydrophobicity [138]; however, blending with other polymers and surface modifications are done to optimize its properties. As an example, Kao *et al.* fabricated 3D PLA-printed scaffolds coated with polydopamine to enhance the adhesion and proliferation of human adipose–derived stem cells [139].

Alizadeh-Osgouei *et al.* utilized fused deposition modeling technology to prepare gyroid scaffolds of PLA to repair large bone defects. Scaffolds showed porosity ranging from 86.1 ± 1.4 to 90.3 ± 0.4% and suitable mechanical strength comparable to those of natural cancellous bone [140].

Mohandesnezhad *et al.* synthesized PCL/PLA electrospun hybrid nanofiber loaded with nanohydroxy apatite and zeolite for dental tissue regeneration. Nanohydroxyapatite and zeolite were synthesized by the hydrothermal method; following, the hybrid nanofiber was fabricated by electrospinning technique. Scaffolds showed enhanced viability and adhesion on human dental pulp-derived stem cells after 1, 7, and 14 days [141].

Gangolphe *et al.* fabricated microstructured electrospun scaffolds of PLA**-**based-copolymers with suitable mechanical properties and high cellular affinity for soft tissue engineering [142]. PLA electrospun mats coated with kefiran were prepared in one study for skin tissue engineering and evaluated by FTIR and SEM to assure the development of a thin kefiran coat wrapped on the fibers. Cell culture assays demonstrated improved embryonic fibroblast cells’ proliferation and induced collagen production [143].

The combination of PLA with chitosan HCl was highlighted in one study, where the authors succeeded to fabricate bio-adhesive hybridized nanoparticles loaded with etoricoxib which were bi-functioning as an anti-inflammatory as well as a bone re-building medicine. The constructed nanoparticles possessed a small particle size of around 500 nm with high entrapment efficiency > 90% “entrapment efficiency is a significant parameter in the assessment of nano-drug delivery systems as it reflects the capacity of the system to enclose the drug [144].” By examining the nanoparticles on MC3T3-E1 normal bone cell line, the ALP activity besides the calcium ions deposition was boosted, indicating the preparation of dual acting approach for the treatment of bone disorders [145].

Table II presents extra studies about the use of the aforesaid synthetic biomaterials.

**Table II:** A Summary of the Utilization of Synthetic Biomaterials for Tissue Engineering Purposes

Drug	Composition	Fabricated dosage form	Targeted tissue and application	Key findings	References
Raloxifene hydrochloride	PLGA/liquid lipid (Maisine®)	*In-*situ forming implant	Bone tissue engineering	• Maisine decreased the burst release of the loaded drug • The prepared implants showed a sustained drug release for 55 days with minimal burst release ranging between 15–20% • The implant possessed a solidification time of around 15 min and a flow rate of 2.12 ± 0.02 mL/min • It showed a porous structure after incubation for one week in the release medium • *In-vivo* evaluation in rats signified the proper bone regeneration with the formation of well-organized bone tissues after 12 weeks of implantation	[146]
Thymosin β-4	PLGA/PLA	Nanofiber/microfiber hybrid yarns	Tendon tissue graft	• *In vitro* release of thymosin β-4 in PBS at 37°C showed a sustained profile for 28 days • This hybrid yarn displayed a nanofibrous structure similar to the ultrastructure of natural tendon tissues • It enhanced the human adipose-derived mesenchymal stem cells**’** growth, proliferation, expression of tendon-specific markers, and collagen deposition • It proved the superiority of the thymosin β-4 loaded hybrid yarn in promoting tendon tissue regeneration compared to PLA microfiber yarns	[147]
Penicillin/ streptomycin	PCL/chitin-lignin/ poly (glycerol sebacate)	Scaffolds made of core–shell fibers	Wound healing	• PCL-coated hybrid fibers had a much longer shelf life and provided sustainable drug release • Penicillin and streptomycin were added to evaluate the effectiveness of the fabricated dressing *versus* *Staphylococcus aureus* and *Escherichia coli*, results showed a great antibacterial effect due to the controlled drug release • Cytocompatibility was assured on bone marrow-derived mesenchymal stem cells for both drug-loaded and control scaffolds [148]	[148]
Dexamethasone	PLA/multiwall carbon nanotubes/PEG	Electrospun nanofibers	Bone tissue engineering	• *In vitro* release in PBS at 37°C revealed that increasing PEG ratios lead to **a** faster drug release pattern where the lowest and the highest ratios exhibited percentage of drug release > 4% and 25% after 9 h, respectively • Multiwalled carbon nanotubes also aided in the homogenous distribution of dexamethasone in the scaffold • The nanofibers were sterilized by UV radiation for 1 h and then incubated with rat bone marrow stromal cells where improved cellular adhesion, proliferation**,** and continuous matrix mineralization via calcium deposition were detected after 21 days for all tested scaffolds • The calcium deposition ranged from 4.5 to 12 µg for all scaffolds except for those containing the highest ratio of PEG which resulted in the deposition of 2.8 µg calcium only	[149]

### Smart Polymers (Stimuli-Responsive)

Smart biomaterials are substances that can reversibly adjust their properties as a response to signals in the surrounding environment. The stimulus to their change could be chemical as the change in pH, or physical such as exposure to light, temperature, electric or magnetic fields; also, they could be stimulated by the action of enzymes (biological stimuli) [150–153]. Native body tissues are in continuous morphological changes in response to the surrounding tissue microenvironment, in the same manner, the use of intelligent materials results in the production of dynamic 3D structures that change as a response to external or internal stimuli. On the other hand, conventional scaffolds cannot transform after fabrication. Also, the use of smart biomaterials leads to a reversible control over the properties of ECM so they can modulate the feedback mechanisms between the cells and the surrounding microenvironment [154, 155].

Nevertheless, stimuli-responsive approaches still need more investigations before being clinically implemented as some systems fail to maintain a correct responsive drug release; for example, pH-dependent scaffolds should swell only as a response to the change in the pH. However, they may mistakenly respond to the moisture change in the wound site leading to non-controlled drug release. Moreover, their mechanical properties are not suitable for all applications as they may suffer from premature degradation and a consequent sudden drug release. Regarding enzyme-controlled biomaterials, although they successfully control the drug release, the elevated enzyme levels may increase the incidence of the exposure of biomolecules to enzymatic attacks. Also, achieving a well-controlled drug release is not easy as all smart materials suffer from an initial burst release when the specific stimulus is present so attaining a specific dosage at a specific time is difficult [156]. The literature includes several examples highlighting the use of smart polymers in the field of tissue engineering as briefly illustrated in Table III.

**Table III:** An Overview on the Use of Smart Polymers in the Tissue Engineering Field

Drug	Smart polymer	Modifications done to the smart polymer	Aim	Stimuli	Key findings	References
Kartogenin	Chitosan	Surface modification to chitosan by N-(β maleimidopropyloxy) succinimide ester Treatment of chitosan with β-Glycerophosphate	*In situ* forming hydrogel for cartilage tissue engineering	Temperature responsive	• The hydrogel was injected non-invasively at the defect • The hydrogel was formed at 37°C within minutes and possessed a suitable shear modulus of 78 ± 5 kPa • It possessed sustained drug release for 40 days • Enhanced chondrogenic differentiation of human adipose mesenchymal stem cells was achieved	[157]
Amikacin Naproxen (preloaded into micelles)	Sodium alginate	Phenylboronic acid was grafted in the side chain of alginate	Inflammation-responsive injectable hydrogel for wound healing	pH and reactive oxygen species responsive	• The hydrogel reduced the TNF-α levels (pro-inflammatory cytokine) about 2.8 times and increased IL-10 levels (anti-inflammatory cytokine) around 2.41 times more than the non-medicated control hydrogel • Sustained naproxen and amikacin release for 24 h in a pH and reactive oxygen species dependant manner • Great antibacterial activity with 90–96% killing ratio against *Staphylococcus aureus* and *Pseudomonas aeruginosa* was obtained • *In vivo* studies in rats revealed huge contraction in wound size treated with medicated hydrogel	[158]
Dexamethasone	Chitosan	Chitosan was coupled with aniline oligomers and mixed with 15 wt% PVA solution	Conductive electrospun nanofibrous mats for tissue engineering	Electro-responsive	• The conductivity value was ≈ 10 − 5 S/cm which was suitable for tissue regeneration • The addition of aniline oligomers enhanced the strength modulus of the mats • The drug release was adjusted according to the need where the electric stimulation resulted in 40% increment in drug release in 40 min compared to unstimulated mats (on-demand drug release) • The mats with the least concentration of oligoaniline demonstrated good cytocompatibility when tested on mesenchymal stem cells due to the presence of biocompatible chitosan	[159]

**Fig. 2:**
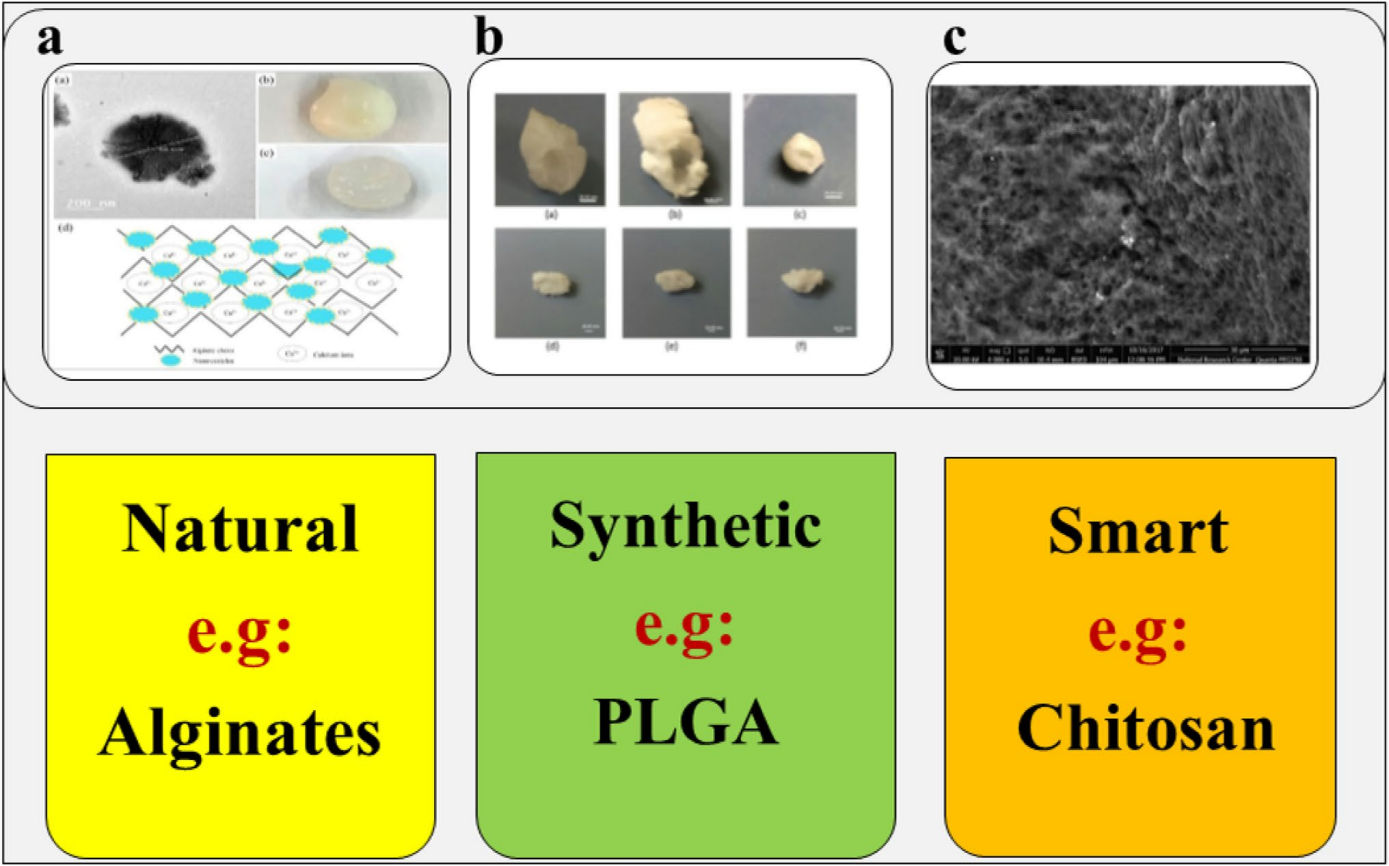
Illustrative diagram of the application of different types of biomaterials in the tissue engineering field. Biomaterials can be natural, synthetic, or smart. **a** Egg-box structure due to interaction between sodium alginate and divalent calcium ions, reprinted from reference [43], with permission from Elsevier. **b** PLGA implants prepared by solvent-induced phase inversion technique, reprinted from reference [146] with permission from Elsevier. **c** SEM of plain *in situ* forming chitosan implants showing its porous structure. The sol–gel transition occurred at 37°C. Reprinted from reference [152], with permission from Elsevier

A brief illustrative overview of the advantages of different biomaterials used in the field of tissue engineering is described in Fig. 2.

### Bioactive Mineral Fillers

The use of different metals in bone and dental tissue engineering is one of the oldest approaches as they possess perfect mechanical and physical properties, they enhance cell proliferation and improve the scaffolds features [108, 160, 161]. Following is a brief discussion about the use of different metals in literature.

#### Silica

Silicon (Si) is one of the most essential trace elements used in bone tissue engineering. Silica nanoparticles induce osteoblasts’ proliferation, mineralization, and differentiation. It has been reported that smaller silica nanoparticles having a size between 50 and 100 nm possess superior osteoinductive activity than the ultrasmall and larger ones [162].

In another study, the authors managed to fabricate hybrid pellets via* the* wet granulation extrusion-spheronization method which were made from two types of amorphous silica; bioglass and doxycycline-loaded mesoporous silica MCM-41 for tissue regenerative applications. Both types of silica were prepared by a sol–gel method followed by the adsorption of doxycycline onto MCM-41, where 73.2 ± 1.6 mg of doxycycline was adsorped per gram of silica. Pellets were evaluated for physical properties and they showed a yield of 78 ± 3%, hardness of 5.5 ± 1.3 N, friability equal to 1.1 ± 0.3%, and drug content value of 91.2 ± 3.5% as well as average size ranging between 1.0 and 1.6 mm. *In vitro* drug release demonstrated biphasic release with 44% of the drug released on the first day followed by a zero-order controlled release for 19 days. Assessment of the mineralization ability of the pellets was evaluated in SBF where a biomimetic apatite layer was formed on the surface of the pellets which was confirmed by SEM with energy dispersive x-ray analysis (SEM–EDX), x-ray diffraction**,** and FT-IR methods. Moreover, the fabricated pellets possessed excellent antibacterial potency *versus* *Staphylococcus aureus* revealed after 7 days besides they showed 100% viability as well as enhanced cellular proliferation when tested on human osteoblasts presenting after 3 days of incubation. The obtained results indicated the success and the safety of the preparation method [163]. Further *in vivo* studies were needed to approve these pellets for bone tissue engineering.

Nekounam *et al.* fabricated electrospun carbon nanofibers composites loaded with silica nanoparticles at different concentrations (1%, 5%, 10%) conducting the electrospinning technique and thermal treatments. SEM of the composite nanofibers demonstrated that the addition of silica nanoparticles increased the diameter of the nanofibers as they were embedded inside the fiber matrix and distributed on the surface of the nanofibers as well. The treatment of the fibers with hydrophilic silica nanoparticles enhanced the hydrophilicity of the fabricated fibers which was indicated by the lowering of the measured contact angle of the composite nanofibers compared to the plain ones. The obtained contact angles were 120.3° *versus* 81° for the aforementioned fibers, respectively. The hydrophilicity of the composites is an essential trait for cellular interactions. Cell line studies on bone osteosarcoma cells (MG-63) indicated the safety of the tested nanofiber composites with 100% viability as well as low concentrations of the detected LDH (< 8%) after incubation for 72 h. Moreover, the cellular proliferation was superior with composite nanofibers than with plain ones, especially with 5 and 10% silica nanoparticles concentration [162]. This study presented electrospun carbon nanofibers composites as a valuable component for bone tissue engineering. Further investigations on drug-loaded nanofibers and evaluation of the *in vivo* behavior of the composites should be considered.

Yu *et al.* produced thermo-responsive covalently-crosslinked composite hydrogels made of chitosan/silk fibroin/amino-functionalized mesoporous silica nanoparticles. Amino-functionalization of silica aided in the crosslinking between the hydrogel components by genipin. The composite hydrogels containing amino-functionalized mesoporous silica nanoparticles possessed satisfactory injectability with rapid solidification in less than 640 s after getting in contact with aqueous fluids. The prepared composite hydrogel displayed good mechanical strength, and elasticity as well as the sustained release of bioactive Si ions for 21 days where the amount of Si released was 50–60 µg/mL after 3 weeks which was sufficient to promote osteogenesis. Cell viability testing by seeding MC3T3-E1 cells onto the hydrogels for 7 days revealed enhancement of cell proliferation and DNA formation signifying the cytocompatibility of the used hydrogels. ALP activity, type-1 collagen formation, and calcium deposition were higher in the hydrogels containing amino-functionalized mesoporous silica nanoparticles compared to the hydrogels lacking its presence proving the osteogenic effect of the release of Si ions [164].

#### Titanium

Titanium (Ti) is widely used in bone implants due to several reasons; (i) Ti implants are considered the ideal choice for hard tissue replacement as they possess mechanical properties resembling those of natural bone tissues [165]; (ii) it *can* create a permanent bond to the bone via osseointegration; (iii) it enhances cell attachment and proliferation; (iv) it improves the mechanical properties of the scaffold by their characteristic load-bearing support ability [166]; and (v) it has been reported to exert an antibacterial effect to the implant [82, 167].

Clainche *et al.* constructed mechano-bactericidal titanium surfaces for bone regeneration applications. They were prepared by different techniques; plasma etching and hydrothermal treatment. The fabricated surfaces produced by plasma etching resulted in the formation of micro-scale two-tier hierarchical topography which decreased the bacterial attachment and led to the bacterial rupture. While the thermally-treated ones produced sharp nanosheets that physically killed the bacteria by cutting their cell membranes. Both surfaces induced adhesion, growth, and proliferation of human adipose-derived mesenchymal stem cells which was indicated by the enhanced ALP activity (≈0.03 U/of μg protein) and calcium deposition (≈0.4 alizarin red absorbance) after incubation for 21 days. The fabricated Ti surfaces promoted osteogenesis without the need to add external growth factors and exhibited bactericidal activity presenting it as a potential key for successful reconstructive surgeries decreasing the liability of nosocomial infections [168].

Deng *et al.* fabricated four types of porous Ti alloy scaffolds (coded as DIA, TC, CIR, CU) designed and selected by computer-aided design software and prepared using selective laser melting. All scaffolds showed similar porosities ranging from 64.8 to 65.3%, pore sizes from 648 to 678 µm, elastic modulus varying between 1.9 and 4.2 GPa, and a yield strength matching the host bone tissues. The authors evaluated the *in vivo* behavior of the fabricated scaffolds by their implantation into the surgically induced defect in the distal femur of New Zealand Albino rabbits. Micro-CT examination for rabbit femurs at 6 weeks signified the gradual formation of new bone tissues with the superiority of one of these alloys (DIA) in promoting bone regeneration as it possessed a porous structure similar to those of trabecular bone tissues. The same results were obtained by the histological examination of femoral condyle samples at the same time intervals which assured the progressive bone tissue growth by the four structures and the superiority of DIA type [169].

One study addressed the coating of Ti metal implants with fibrous composites made from polyvinyl alcohol/hydroxyapatite/folic acid and loaded with methotrexate. The fibrous composites were prepared by electrospinning and applied for bone regeneration purposes. The fibrous composite showed an average diameter of 19 nm**,** as well as high mechanical strength equal to 9660 Pa which was greater than that of plain hydroxyapatite (4965 Pa). Cytocompatibility studies of the prepared composites on human bone marrow-derived stem cells signified their safety which was reflected by enhanced cell growth and proliferation, while it showed cytotoxicity when tested on A459 cells (adenocarcinoma human alveolar basal epithelial cells). This study presented the fabricated fibrous composite as a good choice for osteosarcoma-diseased bone regeneration, but further *in vivo* and clinical studies are needed [170].

In a published study, the authors addressed the main problem which is poor cellular adhesion and fusion on the implant interface. Implants made from nanohydroxyapatite/polyamide 66 blends (which have been used clinically in China for more than 16 years) were treated by plasma-sprayed titanium technique aiming at improving bone cell fusion at the implant interface. Examination with SEM revealed that the plasma-sprayed titanium layer was uniformly distributed along the implant. Additionally, the fabricated implants showed irregular porous surfaces which enhanced the adhesion of osteoblasts and hence a consequent improvement in bone growth. The fabricated implants proved their success when surgically implanted in the induced bone defects in New Zealand Albino rabbits. This success was obvious when 3D micro-CT imaging was performed where it demonstrated great bone formation around the implant with high bone/tissue volume as well as a high trabecular number [171]. This study is considered a great success in bone restoration applications; however, further studies on larger animals with different sized defects should be tried.

#### Niobium

Niobium pentoxide (Nb_2_O_5_) is the oxide form of niobium metal that is used to augment bone tissues. Its use has emerged recently in bone and dental applications. When it comes in contact with saliva, it can induce hydroxyapatite-like crystal growth [172].

Siqueira *et al*. fabricated 3D interconnected porous scaffolds composed of a modified 45S bioactive glass (45 wt% SiO2, 24.5 wt% CaO, 24.5 wt% Na_2_O**,** and 6 wt% P_2_O_5_) where 10% of SiO_2_ in the glass matrix was replaced by niobium pentaoxide. The scaffolds were prepared by gel casting method and possessed a pore size of 100–500 µm, porosity of 89%, and compressive strength equivalent to 0.18 ± 0.03 MPa. The scaffolds showed a similar structure to trabecular spongy bone tissues, *in vitro* evaluation of the prepared scaffold on human osteoblasts MG-63 cells showed good cytocompatibility after 2 and 6 days of incubation and high ALP activity after 48 h of incubation with the cells. The results demonstrated the significant role of Nb in promoting cell proliferation and viability, introducing Nb as a therapeutic ion for bone tissue regeneration [173].

Another study demonstrated the preparation of Ti/Nb alloys with varying Nb contents (0–45 wt.%) using pure Ti and Nb powders via a selective laser melting apparatus. The presence of Nb enhanced the *in vitro* osteogenic ability of the alloy. 3D scaffolds of Ti/25Nb were tested by implantation in the femur of New Zealand Albino rabbits, micro-CT and histological examination revealed that Nb addition accelerated bone healing compared to pure Ti [174].

### Modern Technologies and Recent Applications

**Fig. 3:**
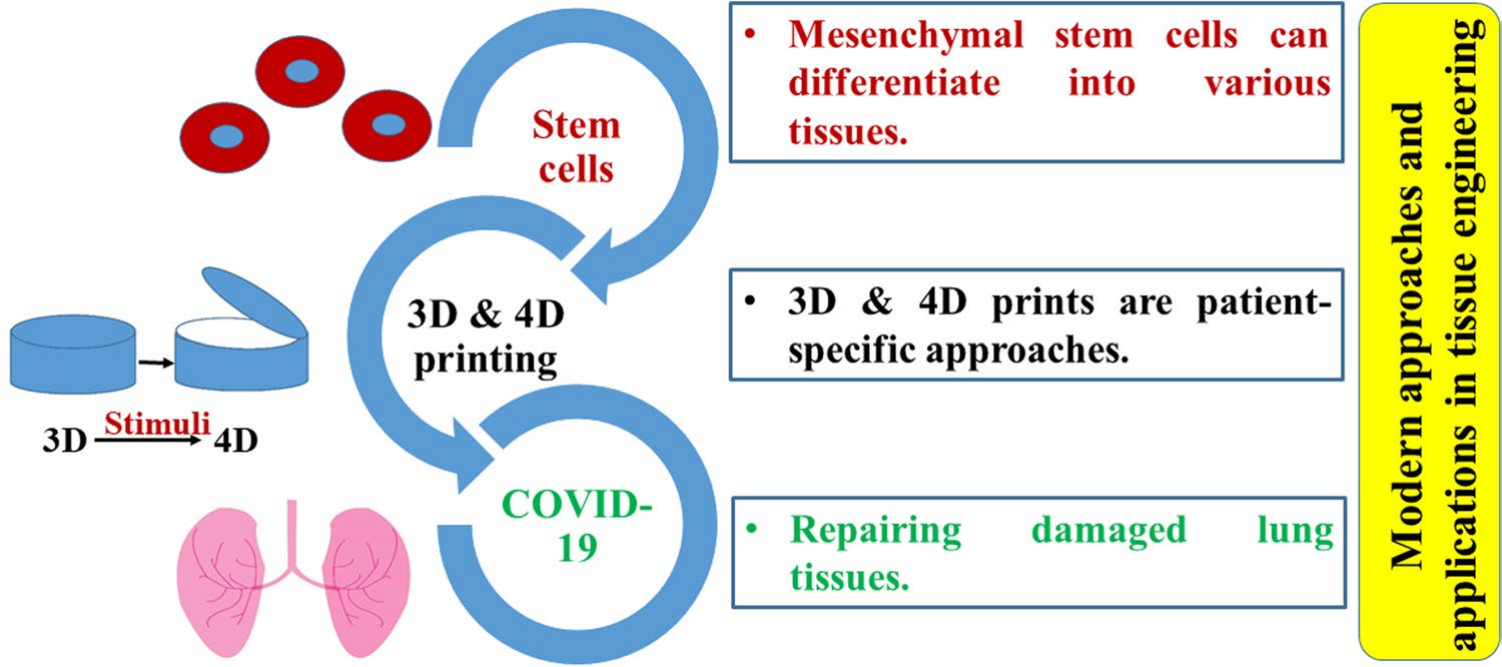
Schematic illustration representing some examples of the insightful strategies for modern tissue engineering approaches

Modern approaches are applied to enhance the outcomes in the tissue engineering field. This review focuses on three approaches as briefly demonstrated in Fig. 3.

#### Stem Cells

Blending the use of stem cells in tissue engineering applications will defeat hard challenges in tissue regeneration strategies [175] by producing engineered tissue substitutes. Mesenchymal stem cells can differentiate into various tissues, they aid in the treatment of bone defects [176], lung injuries [177], thermal burns, skin wounds [178–180]**,** and other different applications. Transplantation of stem cells alone showed poor therapeutic efficacy due to limited viability and low regenerative capacity; however**,** merging them with different biomaterials and scaffolds can solve these problems [181].

Maged *et al.* fabricated crosslinked-chitosan scaffolds containing rosuvastatin and loaded them with mesenchymal stem cells for wound healing. Scaffolds showed good porosity, sustained drug release for 60 h**,** and enhanced human fibroblasts’ cell proliferation. *In vivo* study in Albino rats proved the superiority of mesenchymal stem cells loaded scaffolds over plain ones in promoting cell proliferation and wound closure, moreover, histopathological examination signified stem cells loaded scaffolds in promoting the normal distribution of collagen in the epidermal layer. This study proved the significant effect of stem cells in promoting tissue rejuvenation [182]

#### 3D and 4D Printing

3D printing is one of the most applied techniques in tissue engineering nowadays. This technology depends on printing 3D constructs with high precision, it works through a layer-by-layer addition of different materials. It is considered a cost-effective approach for the production of patient-specific implants according to the shape of the defect by the use of computer-aided designs. Various techniques of 3D printing are applied in the tissue engineering field including fused deposition modeling, selective laser sintering, inkjet-based 3D printing, stereolithography**,** and pressure-assisted micro syringe [183].

Fused-deposition modeling was developed in the 1980s; it is based on melting a thermoplastic polymer and then extruding it through a nozzle to construct a layer-by-layer 3D structure. The advantages of this technique include affordability, simplicity, high speed, and safety as this method is solvent-free. On the other hand, the main disadvantage is the limited number of thermoplastic polymers suitable for usage with proper melt viscosity [129, 184, 185]. Ceretti *et al.* prepared multi-layered scaffolds of PLC for bone tissue engineering using fused-deposition modeling, culture with human foreskin fibroblast demonstrated cytocompatibility of the prepared construct [186].

Selective laser sintering is another approach developed and patented by Carl Deckard and Joe Beaman in 1989. This technique depends on melting a thin layer of powdered material (including ceramics, metals, and thermoplastic polymers) using a laser beam which subsequently fuses into a layer-by-layer 3D structure [187]. The advantage of this technique is the ability to produce large and sophisticated scaffolds, also, it is a safe solvent-free method. However, it produces a rough surface that needs polishing [188]. Gómez-Cerezo *et al.* fabricated porous poly(hydroxybutyrate-co-hydroxyvalerate)/akermanite scaffold for bone tissue engineering using selective laser sintering which showed cell-proliferative activity on human osteoblast cells with improved production of ALP [189].

Although 3D printing shows several advantages, the printed scaffolds are rigid and may not produce the required biological responses. Native body tissues possess continuous morphological changes in response to the surrounding tissue microenvironment, whereas 3D printed scaffolds are static structures that cannot transform after printing.

Four-dimensional (4D) printing was firstly introduced by Skylar Tibbits in 2013 in a speech at the TED conference [190, 191]. 4D printing arises as a recent technology, in which 3D printed scaffolds are made using smart materials that can modify their physical properties over time as a response to an external surrounding stimulus (like temperature, light, electricity, and pH) producing dynamic 3D structures. The changes in the static 3D scaffolds are considered the 4^th^ dimension [35, 192–195]. The major differences between 3 and 4D printing techniques are the feed and the instrument design. In the case of 4D printing, stimuli-responsive polymers were introduced in the 3D printing feeds; the printed objects are able to act in response to environmental stimuli; also, it requires complicated digital designs that are programmed to consider the change in the shape and size of the scaffold over time [196]. One example of a stimulus–response action is the gradual degradation of the implanted scaffold as a response to the new tissue formation which allows complete tissue restoration [191]. The key advantage of 4D printing over 3D printing is the ability to mimic not only the structure of the tissue but also its dynamic function; therefore, this technique provides a way for the innovation of ideal tissue constructs.

#### Applications in COVID-19 Cases

Tissue engineering has emerged as a solution to address the clinical disorders of the current COVID-19 pandemic through various approaches [197–200]. The first includes the utilization of *in vitro* models to understand the host–pathogen interaction and to screen the efficacy of potential therapeutics [201]. The models include different cell lines such as pulmonary cell lines (e.g. human mesenchymal bronchial tracheal cells and human bronchial epithelial cells) and tissue-engineered human *in vitro* lung models [202]. Furthermore, the use of specific biomaterials to achieve successful vaccination and targeted and controlled drug release would be beneficial in conquering the disease [203].

COVID-19 infection causes severe damage to the lower respiratory system by elucidating severe inflammatory response in the lungs which in some cases requires repairing of the damaged tissues. Moreover, the lack of oxygen in the patient’s blood due to alveolar cell damage is a major concern that requires special attention to revive the damaged lung cells as soon as possible to restore normal alveolar function [204]. Preclinical studies on transplanting exogenous mesenchymal stem cells and their derived exosomes have proved their success in repairing damaged lung tissues and improving survival in animal models owing to their anti-inflammatory and tissue regenerative activity [205–207]. Moreover, implantable airways for humans, application of lung progenitor cells derived from human embryonic stem cells, and using tissue-engineered lung tissues can be promising approaches to rejuvenate the damaged lung cells [208]. Lung tissue engineering is aided by several biomaterials such as PCL, PLGA, PLA, collagen, silk, and elastin [209].

Rezaei *et al.* fabricated 3D printed scaffolds made from chitosan/PCL bioinks for lung tissue engineering as a hopeful approach to assess and treat COVID-19 respiratory complications. The scaffolds showed a smooth uniform morphology proving the good dispersion of chitosan and PCL in each other with 55% mean porosity, mean diameter of printed strands was 360 µm, and suitable mechanical and degradation properties. The swelling capacity of the scaffolds ranged between 13 and 21% after 72 h of incubation which indicated their suitability to exchange nutrients and wastes during cell growth. Cytocompatibility assessment on MRC-5 cells demonstrated the safety of the fabricated scaffolds. SEM images proved proper cell adhesion on the scaffolds where the cells started to spread through its surface over time. This research presented a promising 3D construct that needs extra studies and investigations to be approved for COVID-19 assessment and treatment [210].

## Conclusions

Tissue engineering has emerged as a milestone in accelerating tissue healing and promoting the quality of life of patients with organ defects. It is considered a golden alternative for organ transplantation. Many options are provided by the utilization of different biomaterials and their blends either from natural (e.g. gelatin, collagen celluloses, and zein) or synthetic (e.g. PLGA, PCL, and polyurethanes) origins. Generally speaking, natural polymers are more favored in use than synthetic ones, specifically, the use of plant-derived biopolymers which is highly encouraged than animal-derived ones as they lack ethical issues, are extracted easily, and are of lower cost. This review discusses the recent approaches reported by different authors employing various biopolymers and their composites for divergent tissue regeneration applications. The choice of a specific biomaterial depends on the requirements of the targeted tissue; however, the use of composite materials opens the way to finely adjust the properties of the used scaffolds. Furthermore, the utilization of bioactive mineral fillers (e.g. silica, titanium, and niobium pentoxide) in tissue regeneration represents a valuable approach to get the advantage of their favorable mechanical and tissue regenerative properties. Interestingly, modern technologies arise in the tissue engineering field where the usage of stem cells has been extensively studied as they can differentiate into various tissues, another cost-effective modern approach included 3D and 4D printing of scaffolds, while the most recent application of tissue engineering is the implication of different strategies to understand and treat COVID-19 infections. Tissue engineering has indeed been studied extensively by many authors; nevertheless, clinical applications are still limited and need more investigation.

## Data Availability

Data is available within the article.
